# Purified malignant mammary epithelial cells maintain hormone responsiveness in culture

**DOI:** 10.1038/sj.bjc.6600866

**Published:** 2003-04-01

**Authors:** M S Kothari, S Ali, L Buluwela, N Livni, S Shousha, H D Sinnett, R Vashisht, P Thorpe, S Van Noorden, R C Coombes, M J Slade

**Affiliations:** 1 Department of Cancer Cell Biology, Imperial College, Du Cane Road, London W12 ONN, UK; 2 Department of Histopathology, Charing Cross Hospital, Fulham Palace Road, London W6 8RF, UK; 3 Department of Surgery, Charing Cross Hospital, Fulham Palace Road, London W6 8RF, UK; 4 Department of Surgery, West Middlesex University Hospital, London TW7 6AF, UK; 5 Department of Histopathology, West Middlesex University Hospital, London TW7 6AF, UK; 6 Department of Histopathology, Imperial College, Du Cane Road, London W12 ONN, UK

**Keywords:** primary cells, oestrogen receptor, hormone responsiveness

## Abstract

Currently, the therapy for breast cancer is determined by immunohistochemical staining of the primary tumour for oestrogen receptor alpha (ER*α*). However, a proportion of ER*α*-positive patients fail to respond to tamoxifen and a proportion of ER*α*-negative patients show response. Here, we describe a novel procedure for the purification of malignant breast epithelial cells in an attempt to identify these patients at an early stage. Using this procedure, we are able to purify malignant cells to >90% purity as determined by immunohistochemical staining, cytology and fluorescent *in situ* hybridisation (FISH). While the malignant cells can be maintained in culture they do not proliferate in contrast to purified breast epithelial cells from reduction mammoplasties. Moreover, ER*α* and progesterone receptor (PR) expression is maintained in malignant cells, whereas normal epithelial cells rapidly lose ER*α* and PR. Functional studies were performed on the separated malignant cells in terms of their response to oestradiol and tamoxifen. Four out of the seven ER*α*-positive tumours showed a significant reduction in cell numbers after tamoxifen treatment compared to oestradiol, ER*α* negative tumours failed to show a response. We conclude that (a) it is possible to purify and maintain breast cancer cells for a sufficient period to permit functional studies and (b) ER*α* is retained in culture facilitating the use of these cells in studies of the mechanism of endocrine response and resistance *in vitro*.

The human mammary gland is comprised of two epithelial cell components, namely luminal epithelial and myoepithelial cells, both of which can be purified by immunoaffinity techniques that exploit differences in cell surface protein expression (Clarke *et al*, 1994; Gomm *et al*, 1995,1997a; Kamalati *et al*, 1999; Niranjan *et al*, 1995; Page *et al*, 1999; Slade *et al*, 1999). Using such protocols, it has been possible to further study and highlight the responses of these two cell types to growth and morphogenic signals, thereby gaining further insight into the functionality of these cells in normal mammary gland function.

It is thought that luminal epithelial cells are the main precursor cell population from which breast cancers arise, although gene expression studies indicate that there may be myoepithelial gene expression signature in a significant number of such tumours (Perou *et al*, 1999,^2000^). Nevertheless, comparison of tumour and normal cells highlight oestrogen receptor alpha (ER*α*) expression in breast cancer, a protein found to be expressed in approximately 20% of all cells in the normal mammary gland (Russo *et al*, 1999), and 87% of ER-positive cells are luminal epithelial cells or in an intermediate position in the duct wall (Petersen *et al*, 1987). ER*α* is expressed in 50–70% of all breast cancers, where endocrine therapy directed at inhibiting ER*α* function is used as a treatment following surgery. From this, and through observations in animal models it is clear that oestrogen is an important growth and/or survival factor in breast cancer. However, the response of primary human breast tumour cells to oestrogens and antioestrogens remains poorly studied. Although there are a number of ER*α*-positive breast cancer cell lines, these may not be the best vehicle for studying *in vivo* response. These problems have prompted us to evaluate the potential for immunoaffinity purification to characterise primary human breast tumour cells. In doing this, we sought to modify our original protocols for the purification of normal mammary epithelial cells so that they can be applied to both the normal and malignant mammary gland. By exploiting the expression of epithelial cell adhesion molecule (Ep-CAM) antigen, found on the surface of primary luminal epithelial and tumour cells, we have been able to obtain near homogeneity in purifications for both cell types. Surprisingly, we find that even though both tumour and normal cells are purified using similar protocols, ER*α* expression in primary normal cells is unstable and is rapidly attenuated. By contrast, ER*α* expression is stable and can be maintained in cultures of purified malignant epithelial cells. Finally we show that, under conditions that stimulate growth of the normal cells purified, tumour cells do not proliferate and remain in this quiescent state, even following treatment with oestrogen.

## MATERIALS AND METHODS

### Tissues used

Fresh tissue was obtained from 82 cases of primary breast carcinoma (Table 1

**Table 1:** Patient characteristics (*n*=82)

	**Number**	**Percentage**
*Pathological size (cm)*		
<2	32	39
>2	50	61
		
ER+/PR+	57	69
ER+/PR−	8	10
ER−/PR+	3	4
ER−/PR−	14	17
		
*Histology*		
Invasive ductal	61	74
Other/mixed invasive	21	26
		
*Nodal status*		
Positive	42	51
Negative	40	49
		
*Grades*		
III	28	34
II	45	55
I	9	11

This shows the total number of patients from whom tumours were obtained during the study.

) and six cases of reduction mammoplasty, which on subsequent histopathological examination showed no signs of malignancy or other abnormality. Of the 82 primary breast carcinomas (Table 1), 31 were used for development of the purification protocol/culture conditions and 19 resulted in insufficient cells for any studies. Of the remaining 32 samples, 18 were examined cytologically and of these five were also analysed by fluorescent *in situ* hybridisation (FISH) and seven were stained for ER*α*/PR. Four samples were used in thymidine incorporation, seven in the oestrogen/tamoxifen studies and the remaining three were used for adherence studies. All patients were treated at Charing Cross and West Middlesex Hospitals, London, and gave written, informed consent as required by the local Ethics Review Board.

### Purification of normal human mammary luminal epithelial cells

Cells were purified from breast tissue from reduction mammoplasties using a modification of procedures described previously (Gomm *et al*, 1995; Slade *et al*, 1999). Briefly, breast tissue was minced and digested overnight at 37°C with type IA collagenase (1 mg ml^−1^) in RPMI-1640 plus 5% foetal calf serum (FCS) and 2 mM L-glutamine, 100 U ml^−1^ penicillin, 0.1 mg ml^−1^ streptomycin, 50 U ml^−1^ polymixin B, 2.5 mg ml^−1^ amphotericin B. Following digestion, the fat was decanted off and the remaining organoids and cells were washed three times in media. Organoids were left to settle out for 20 min, the supernatants were removed, and resuspended in RPMI-1640 containing 1% FCS. After two further rounds of sedimentation, they were digested with trypsin/EDTA (0.05/0.02% in PBS) plus 0.4 mg ml^−1^ DNaseI for 15–30 min at 37°C and the reaction was terminated by the addition of cold RPMI plus 10% FCS. Epithelial cells were immunoaffinity purified using superparamagnetic, polystyrene beads (Dynal Ltd, New Ferry, Wirral, UK) coated with a mouse IgG1 monoclonal antibody (MAb Ber-EP4) specific for two (34 and 39 kDa) glycopolypeptide membrane antigens (Latza *et al*, 1990). Purified cells were subsequently cultured in BCM (DMEM:F-12 (1 : 1), supplemented with 15 mM Hepes, 2 mM L-glutamine, 100 U ml^−1^ penicillin, 0.1 mg ml^−1^ streptomycin, 50 U ml^−1^ polymixin B, 2.5 mg ml^−1^ amphotericin B, 5 mg ml^−1^ insulin, 10 mg ml^−1^ apo-transferrin, 100 mM ethanolamine, 1 mg ml^−1^ hydrocortisone and 10 ng ml^−1^ EGF) containing 10% FCS (Gomm *et al*, 1995,1997b; Slade *et al*, 1999).

### Purification of malignant epithelial cells from primary tumours

The tissue was minced and digested as above. However, the digestion was continued only until predominantly a single-cell suspension was achieved (usually 2–5 h). Undigested material was removed using a 50 *μ*m pore nylon mesh. Tissue remaining on the mesh was subjected to further collagenase digestion (1–3 h) and again filtered through a 50 *μ*m pore nylon mesh. The two filtrates were refiltered with progressively smaller filter sizes (50–28 *μ*m). Under these digestion conditions, the majority of normal epithelial cells remained in intact organoids or as clumps and were removed by the filtration steps. The tumour cells were purified as described above and isolated cells were cultured in BCM with 5% FCS (FCS concentrations from 1 to 10% were investigated and 5% resulted in the highest cell viability after 5 days in culture (data not shown)).

### Characterisation of isolated tumour cells

Purified tumour cells were cytospun and stained with hematoxylin/eosin (H&E) and May Grunwald Giemsa (MGG). The percentage of malignant cells was determined by conventional cytology before purification, after purification and after 7 days in culture following purification.

ER*α* and progesterone receptor (PR) expression was assessed at different time points by immunostaining on cells fixed in Zamboni reagent (Stefanini *et al*, 1967), using anti-ER*α* (MAb NC-ER-6F11 Novocastra, Newcastle, UK) and anti-PR (MAbPR-88, Menarini Diagnostics, Wokingham, UK) according to manufacturers' protocols. Cell purity was determined by staining for cytokeratins 8 and 18 (MAb CAM 5.2, Becton Dickinson, Oxford, UK).

Fluorescent *in situ* hybridisation (FISH) analysis of purified cells was performed as described (Kallioniemi *et al*, 1992), using centromere-specific fluorescent probes (Vysis Inc., Downers Grove, IL, USA) for chromosomes 6, 7 and 12 (spectrum orange), 11, 17 and 18 (spectrum green) (Cajulis and Frias-Hidvegi, 1993; Persons *et al*, 1996). These were used to detect aneusomy according to the manufacturer's instructions. Only cells exhibiting three or more spots were deemed to be showing aneusomy.

In order to assess the viability/adherence characteristics of the purified normal and malignant cells, 1 × 10^5^cells were plated into 96-well plates. Three days postseeding, the medium was removed, the adherent cells were washed twice with PBS and the washings pooled with the medium. The number of viable and nonviable cells were counted using trypan blue. The adherent cells were removed by trypsinisation and counted. This process was repeated after 3 and 6 days. Purified cells from three reduction mammoplasties and three primary tumours were assessed.

### Effects of oestradiol and tamoxifen

1 × 10^5^ cells were plated into 96-well plates. Culture medium was changed on the third day for BCM+5% FCS containing 17*β*-estradiol (E2; 10 nM) or 4-hydroxytamoxifen (4 OHT; 100 nM) or an equal volume of the vehicle, ethanol. Total and viable (trypan blue) cell counts were measured during the time course of the experiment.

## RESULTS

### Optimisation of purification procedure

We have previously described procedures for the purification and culturing of normal human mammary luminal epithelial cells. In order to adapt these techniques to the purification and culturing of tumour cells, we have evaluated the use of Ber-Ep4 in this context. In doing this, we have exploited the differential collagenase treatment of normal mammary organoids compared to primary tumour cell aggregates, which show a markedly greater sensitivity to this process (this step is responsible for eliminating residual normal cell within the preparation). We have been able to show that careful use of collagenase leads to single-cell suspensions of tumour cells with normal cells remaining in aggregates large enough to be removed by conventional filtration. Prior to incubation with the immunolabelled beads, the median cell number was 1.5 × 10^6^ (range: 1 × 10^5^–7 × 10^6^). After purification, the median cell number was 3 × 10^5^ (range: 0.3 × 10^5^–6 × 10^6^). We also evaluated other parameters, including the ratio of beads to cells required to obtain tumour cell preparations of high purity. The number of beads: cell ratio was maximally 10 : 1 when there were <10^5^ cells but apart from this the optimised ratio was 3 : 1, which we found to result in a high degree of purity without the cells being completely coated with beads.

### Characterisation of isolated tumour cells

Following collagenase digestion, the cells were assessed by cytology ( Table 2A

**Table 2A:** Percentage malignancy as determined by cytology before and after (day 0) immunobead selection and after 7 days in culture

	**% Malignancy cytology**		
	**Pre-beads**	**Day 0**	**Day 7**
T1	69	97	95
T2	65	97	96
T3	72	97	96
T4	88	98	95
T5	61	97	99
T6	87	100	97

) and immunostaining for cytokeratins 8 and 18 expression (Figure 1A, C

**Figure 1:**
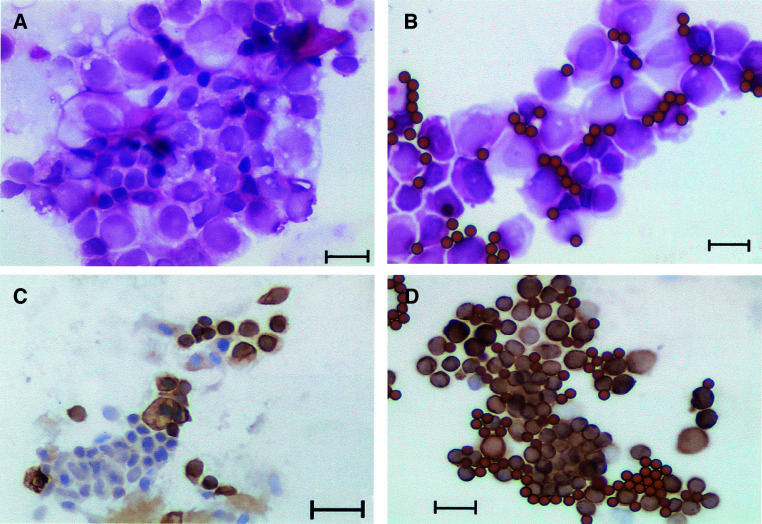
(**A**) H&E staining of cells from a primary tumour before purification, (**B**) H&E staining of cells from a primary tumour after purification, (**C**) cytokeratin 8 and 18 staining of cells from a primary tumour before purification, (**D**) cytokeratin 8 and 18 staining of cells from a primary tumour after purification (Bar represents 20 *μ*m).

). After immunobead purification, the cells were assessed for purity and >95% of the purified cells showed abnormal cytology, and cytokeratin staining revealed that between 95 and 100% (mean 97%) of cells were positively stained (Figure 1B, D). There appeared to be only 1–5% contamination (mean=2%), mainly because of the presence of degenerate cells and occasional macrophages. (Table 2B

**Table 2B:** Percentage aneusomy at days 0 and 7 following purification as determined by FISH

	**% Aneusomy FISH**													
	**Day 0**							**Day 7**						
	**6**	**7**	**11**	**12**	**17**	**18**	**Total**	**6**	**7**	**11**	**12**	**17**	**18**	**Total**
T2	25	26	75	73	43	88	92	23	–	75	72	–	81	87
T3	–	43	–	73	50	69	92	–	43	–	69	52	72	87
T4	–	44	–	63	74	70	86	62	55	71	–	72	–	91
T5	59	60	79	60	70	68	85	–	–	–	55	–	62	82
T6	65	68	74	–	54	–	86	58	70	78	–	46	–	85

Abnormal cytology was based on the nuclear : cytoplasmic ratio, the presence of nucleoli and the number as compared to cultured normal cells and on the increased presence of chromatin as compared to normal cells. Cell numbers after purification: T2 2.8 × 10^5^, T3 3.6 × 10^5^, T4 2.2 × 10^5^, T5 1.2 × 10^5^ and T6 4.5 × 10^5^.

displays aneusomy using paired chromosome probes).

Immunostaining on a subset of ER*α*- and PR-positive tumours over 9 days showed that these phenotypes were retained throughout the time course studied *in vitro* (Table 3

**Table 3:** ER*α* and PR staining of separated cells from seven primary tumours

	**Day 0**			**Day 3**			**Day 6**			**Day 9**		
**Tumour (ER*α*/PR status)**	**Viable cells**	**ER*α***	**PR**	**Viable cells**	**ER*α***	**PR**	**Viable cells**	**ER*α***	**PR**	**Viable cells**	**ER*α***	**PR**
T1^(+++/+)^	0.7	35.0	ND	0.4	39.6	ND	0.3	37.1	ND	0.2	32.9	ND
T4^(+++/+++)^	2.0	44.0	ND	1.5	27.3	ND	1.2	21.6	ND	1.1	24.1	ND
T5^(+++/+++)^	0.9	28.4	ND	0.7	25.1	ND	0.6	25.4	ND	0.5	24.4	ND
T6^(+++/+)^	4.1	65.0	ND	3.1	56.8	ND	2.6	71.2	ND	2.4	67.7	ND
T7^(+++/+++)^	6.0	25.3	64.4	4.5	30.9	60.4	3.0	30.7	59.2	2.7	28.8	53.6
T8^(++/++)^	26.6	79.5	81.5	21.6	80.2	67.2	14.9	70.2	62.2	13.5	60.1	64.0
T9^(−/−)^	3.2	5.8	3	2.9	5.2	<3	2.0	5.5	<3	ND	ND	ND

The number of viable cells is expressed as x 10^5^. ER*α* and PR staining are expressed as a percentage. ND=not done.

and Figure 2

**Figure 2:**
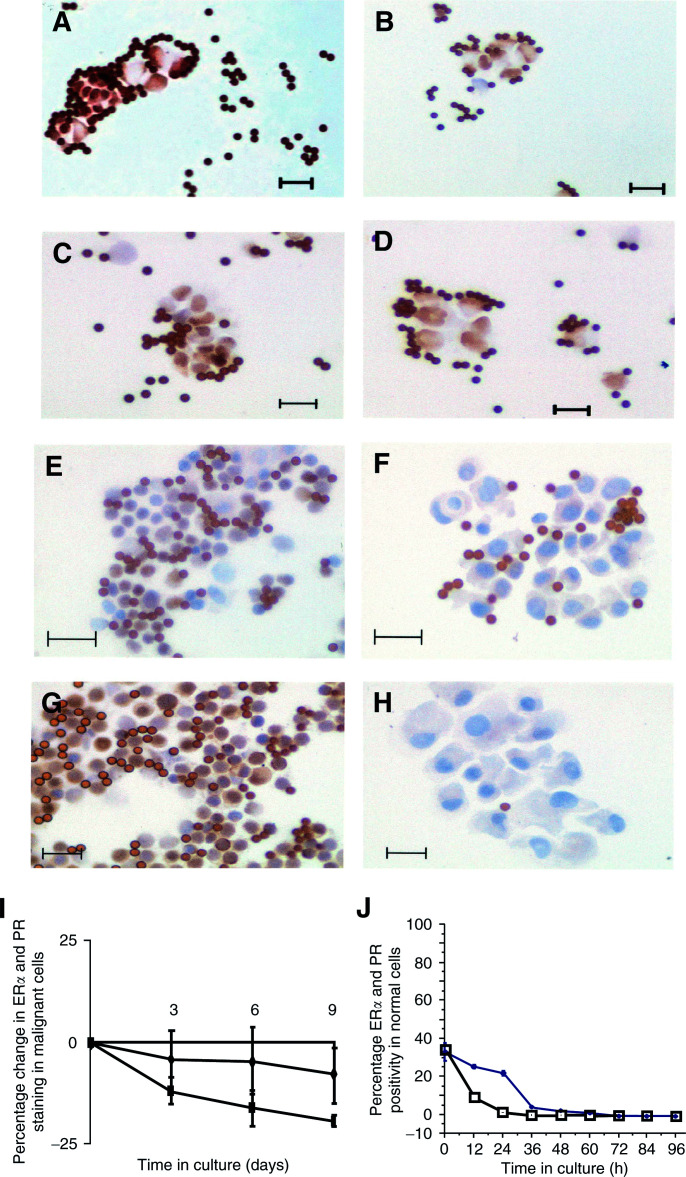
(**A**) ER*α* staining of cells from a primary tumour after immunobead purification (day 0), (**B**) ER*α* staining of cells from a primary tumour (day 7), (**C**) PR staining of cells from a primary tumour after immunobead purification (day 0), (**D**) PR staining of cells from a primary tumour (day 7), (**E**) ER*α* staining of cells from a reduction mammoplasty after immunobead purification (day 0), (**F**) ER*α* staining of cells from a reduction mammoplasty (day 7), (**G**) PR staining of cells from a reduction mammoplasty after immunobead purification (day 0), (**H**) PR staining of cells from a reduction mammoplasty (day 7). The bar on each figure represents 20 *μ*m, (**I**) ER*α* and PR status of primary malignant mammary epithelial cells over 9 days expressed as the percentage change. There was a reduction in the percentage of cells positive for both ER*α* and PR; however, the majority of the malignant cells remained ER*α*- and PR-positive after 9 days in culture (⧫) ER*α* (*n*=6), (▪) PR (*n*=3), and (**J**) ER*α* and PR status of primary normal mammary epithelial cells over 96 h. Both receptors were rapidly lost in the normal cultures, so that after 36 h in culture, all the cells were ER*α* and PR negative (□) ER*α* and (•) PR.

A–D). However, in six normal cultures studied, there was a substantial reduction in expression of ER*α* and PR (Table 3 and Figure 2E–H). When this was examined in more detail, we observed that in fact there was a substantial reduction in ER*α* and PR expression within 24 h and ER*α* expression was not observed beyond this period whereas PR expression was lost in four cultures by 60 h (Figure 2I, J). Although this pattern of staining is not indicative of malignancy it does indicate that the epithelial cells isolated from the primary tumour were abnormal.

In order to confirm that the cells isolated from the primary tumour maintained an abnormal genotype in culture, we carried out FISH on a subset (*n*=5) using probes for six chromosomes and compared this with normal cells purified from reduction mammoplasty specimens (*n*=5). We compared the percentage showing aneusomy using paired chromosome probes before and after 7 days' culture (Table 2B). It can be seen that between 85 and 92% of cells showed aneusomy at day 0 and 82–91% at day 7. The figure for individual probes was 26–88%. No normal breast epithelial cells showed aneusomy.

**Figure 3:**
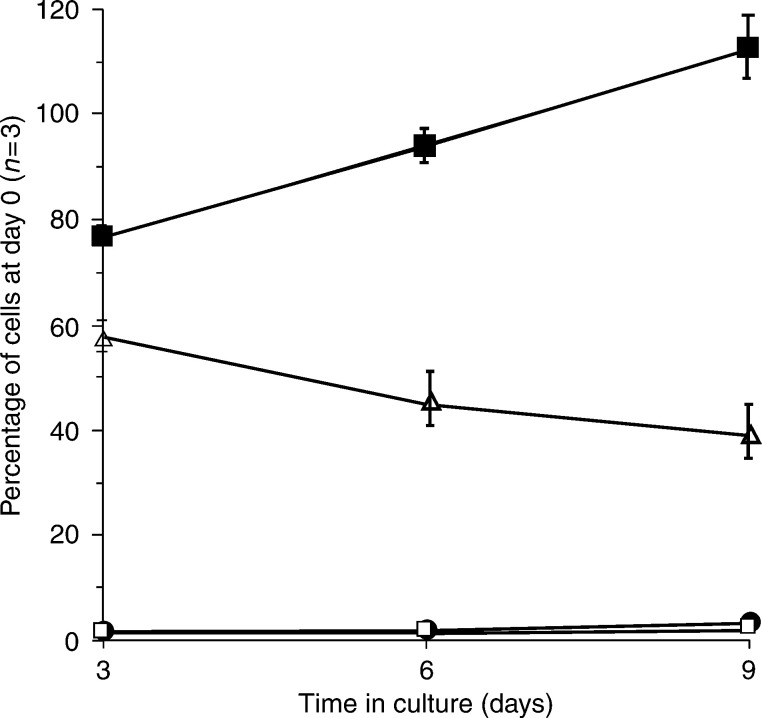
Graph showing the viability of primary normal and malignant mammary epithelial cells over 9 days as determined by trypan blue staining and cell counting. The experiment started 3 days after the cells were seeded in order to allow for cell adherence. The adherent normal epithelial cells (▪) proliferate and hence the increase in numbers after the initial reduction between days 0 and 3. Approximately 60% of the malignant epithelial cells were adherent after 3 days and this fell to approximately 45% 9 days after seeding (▵). The number of floating dead malignant cells (□) and the floating live malignant cells (•) was low throughout the experiment.

We examined the proportion of cells that retained viability and adherence in a subset of samples (*n*=3). Figure 3

shows that, at day 3, more than 50% of cancer cells were viable and adhered to plastic. At day 9, this fell to approximately 40%. In contrast, the normal epithelial cells increased in number, so that the numbers at day 9 were greater than the number initially seeded. There was no evidence of proliferation of the cancer cells, despite the fact that the MTS assay (Promega Inc., Southampton, UK) indicated maintained cell viability (data not shown). In addition, thymidine-uptake measurements showed no uptake over 16 days (data not shown).

### Effects of oestradiol and tamoxifen

We studied the effects of oestradiol (E2) and 4OHT in ER*α*-positive and -negative tumours. As a result of a limited number of cells, studies were limited to two time points. Trypan blue staining determined viable cell numbers. The medium was changed on the third day of culture to E2 and 4OHT-containing medium, thereby allowing sufficient time for the cells to adhere to plastic.

**Figure 4:**
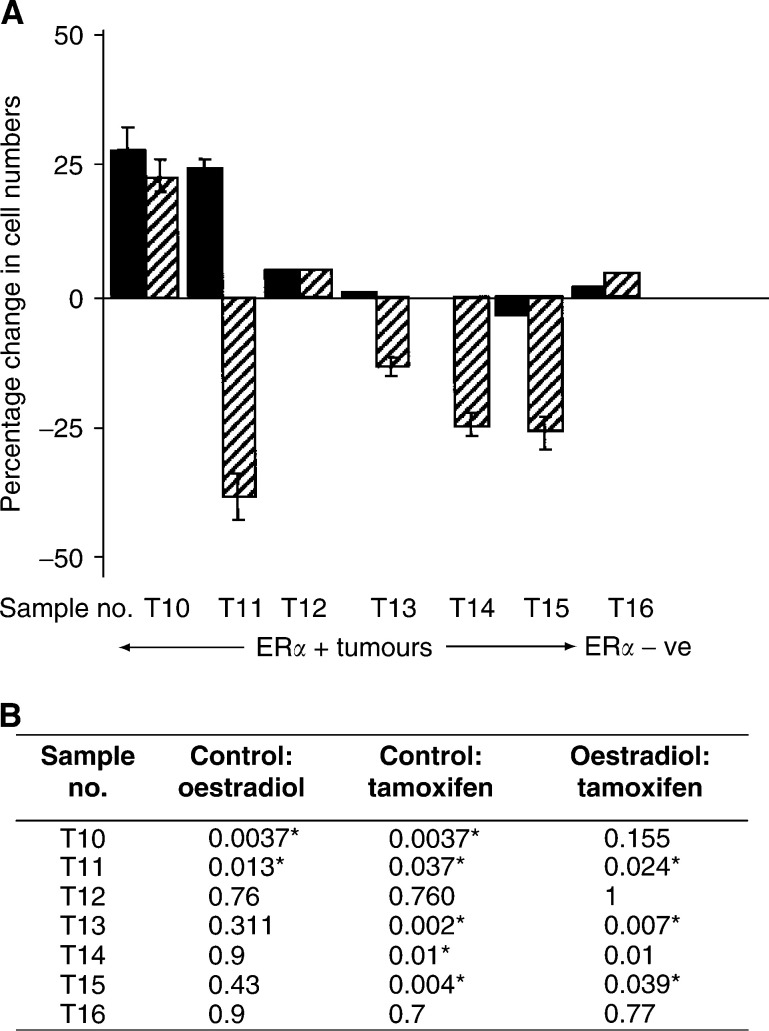
(**A**) The effects of oestradiol and tamoxifen in terms of cell survival on malignant mammary epithelial cells (▪) Oestradiol, (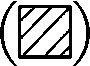) Tamoxifen. (**B**) Table showing the *P*-values for each tumour comparing ethanol control to oestradiol, ethanol control to tamoxifen and estradiol control to tamoxifen. An asterisk indicates a statistically significant difference.

Four (all ER*α* positive) of the seven tumours studied showed significant reduction in cell numbers after tamoxifen treatment compared to oestradiol treatment. The ER*α*-negative tumour failed to show a response (Figure 4A

). The *P*-values were calculated for each tumour comparing ethanol control to oestradiol, ethanol control to tamoxifen and oestradiol control to tamoxifen, and are shown in Figure 4B. We have shown that oestrogen is required for cell survival as primary tumour cells cultured in phenol red-free media + 5% double-stripped serum had a markedly reduced cell viability within 24 h (data not shown). Therefore, residual oestrogen content of the FCS does not explain the lack of proliferation.

## DISCUSSION

We show, in this report, that enzymatic treatment of breast cancer, followed by immunomagnetic breast cell purification, yields extremely pure populations of breast cancer cells, as judged by cytology and FISH. These methods have been developed as a direct extension of our studies of normal breast cell purification (Gomm *et al*, 1995,1997a; Slade *et al*, 1999) and demonstrate the value of immunomagnetic breast purification for both normal and neoplastic breast cells. Other results presented here demonstrate distinct differences in retention of ER*α* expression in normal and cancer cells with normal cells losing ER*α* expression within hours of purification. However, despite loss of ER*α* immunostaining in normal cells, we have evidence of retained ER*α* expression since the normal cells respond to oestrogen exposure by expressing both PR and PS2 (Zhuang *et al*, 2003), both of which are known to be oestrogen-regulated. Further, the cells show an increase in proliferation in response to oestrogen and a decrease following tamoxifen administration. In contrast, ER*α*-positive purified breast cancer cells show a reduction in cell death following estradiol reversed by tamoxifen, but in no case did we observe proliferation of breast cancer cells. It is of interest that no effect of oestrogen was seen in the ER*α*-negative breast cells.

With regard to the observation that ER*α* expression is rapidly attenuated in normal cells, several mechanisms may be relevant. Firstly, growth factor signalling is known to regulate ER*α* expression through unclear mechanisms; the transcription factor AP2*ϒ* is known to be important in regulating ER*α* expression and the levels of AP2*ϒ* are elevated in breast cancer as compared to normal breast (Turner *et al*, 1998). Recent studies (Wijayaratne and McDonnell, 2001) have suggested a link between transcription factor activity and the rate of degradation, with greater activity equating with deceased protein stability. ER*α* is degraded via the 26S proteosome pathway and ubiquitination is influenced by ligands, unliganded receptor having a short half-life. It is hence possible that breast cancer cells, by virtue of their elevated aromatase content, have a higher likelihood of possessing ligand-occupied ER*α* (Wijayaratne and McDonnell, 2001).

Some groups have published protocols for the purification of breast cancer cells. Most remarkable of these have been Spiers *et al* (1998), who used differential centrifugation to purify different cell types and Dairkee *et al* (1997), who used partial enzymatic degradation. However, the methods used cannot exclude contamination from other cell types, particularly normal epithelial cells and fibroblasts. In Spiers' method, overgrowth by normal epithelial cells is suggested by the observation that ER*α* expression was gradually lost over time (Speirs *et al*, 1998), whereas in Dairkee's (1997) method, contamination by fibroblasts required differential trypsinisation. Unlike other groups (Dairkee *et al*, 1997), we have never observed proliferation of the purified neoplastic cells, in contrast to the normal luminal cells, which increased in number in the culture conditions used (Gomm *et al*, 1997a,1997b). Others have published work on transformed normal breast epithelial cells in culture (Yaswen *et al*, 1990,^1992^; Stampfer and Yaswen, 1993) where resemblance to primary cancer cells may be lost.

To overcome some of these problems, we devised an immuno-affinity purification procedure using magnetic beads resulting in a very pure population of epithelial cells, as judged by PCR for luminal epithelial and myoepithelial markers. Our recent studies have demonstrated that these two populations of cells have distinct growth medium requirements in that epithelial cells require FCS, EGF and FGF, whereas myoepithelial cells are growth inhibited by FCS (Gomm *et al*, 1997b). We took these findings into consideration when choosing media for our cells.

In conclusion, we believe that despite the limitations in cell numbers obtained, we have established several characteristics of these cells, including viability, cytogenetics, ER*α*/PR status and response to oestrogen, and that this is a viable methodology for purifying small numbers of primary breast cancer cells, which will be a useful tool in the study of breast cancer biology.
